# Letter to the editor: False-positive results with rapid diagnostic tests (RDT) for dengue

**DOI:** 10.2807/1560-7917.ES.2019.24.21.1900304

**Published:** 2019-05-23

**Authors:** Emilie Javelle, Philippe Gautret, Isabelle Leparc-Goffart

**Affiliations:** 1 Laveran Military Teaching Hospital, IHU-Méditerranée Infection, Aix Marseille Univ, IRD, AP-HM, SSA, VITROME, Marseille, France; 2 IHU-Méditerranée Infection, Aix Marseille Univ, IRD, AP-HM, SSA, VITROME, Marseille, France; 3 IRBA, Unité de virologie, CNR des Arbovirus, Marseille, France; 4 Unité des Virus Emergents, Aix-Marseille Univ, IRD 190, Inserm 1207, IHU Méditerranée Infection, Marseille, France

**Keywords:** dengue, chikungunya, rapid diagnostic test, false positive, Thailand

**To the editor:** In a recent issue of *Eurosurveillance*, Kantele reported a cluster of five chikungunya cases among Finnish travellers to Koh Lanta in Thailand [1]. Two of them had positive rapid diagnostic tests (RDTs) for dengue performed in Thailand, concurrently with detection of chikungunya virus (CHIKV) antibodies, so that a co-infection with dengue virus (DENV) and CHIKV was diagnosed on site. However, after returning to Finland, DENV antibodies were negative at day 11 after the onset of symptoms, while anti-CHIKV IgM and IgG were strongly positive in ELISA (IgG > 2,560). Since a negative dengue serology more than 7 days after the onset of symptoms excludes an acute first or secondary infection [2], co-infections with DENV were finally ruled out in the two travellers.

In recent years, concerns have arisen with respect to the performance of the RDTs for dengue. An international workshop reviewed data about methods in use in 2004 [2]; though the inventory provided at that time may now be obsolete, the workshop’s conclusions remain valid: the ideal diagnostic test for clinical purpose should distinguish between DENV and other flaviviruses, be highly sensitive for all DENV serotypes and give an early positive result in all acute infections, as well as throughout the whole acute phase of the illness. Despite an increasing number of commercially available RDTs, none have reached these requirements yet and their sensibility/specificity varies from one to another.

RDTs for dengue are based on the detection of antigens and/or antibodies. Tests may use recombinant viral envelope glycoproteins of DENV 1, 2, 3 or 4 to detect specific IgM or IgG, or they may use immunoglobulins to detect viral envelope antigens or nonstructural viral proteins such as the NS1 antigen. None is accurate enough to be highly sensitive for the detection of all four DENV serotypes, to correctly diagnose acute primary and secondary infections, and to differentiate between DENV and other flaviviruses such as Japanese encephalitis, West Nile fever, yellow fever and, in particular, the potentially co-circulating Zika virus [3]. Tests detecting antibodies, including those for malaria or flavivirus infections, suffer from a lack of specificity in varied contexts [4], while NS1 detection kits mostly lack sensitivity, sometimes with variation from batch to batch. Thus, viral diagnostic methods that combine antigen and antibody detection have improved testing accuracy.

Aside from the possibility that the RDTs came from a defective batch, there are two main hypotheses to explain the results in the two Finnish travellers. First, the test that was used was a rapid immunochromatographic dengue test that cross-reacts with peptides synthesised during acute CHIKV infection, notably rheumatoid factors (mainly observed with IgM-based RDTs) [5]. Note that cross-reaction with anti-alphavirus antibodies is unlikely. Second, it could be that the RDT false-positive results were due to another flavivirus infection. Indeed, we don’t know if the serological assay for DENV performed in Finland was able to detect IgG against all flaviviruses. According to what was presented in the article, we can assume that the two travellers were not tested for Zika virus co-infection, though it has been circulating at a low but sustained level for at least 16 years in the whole Thai territory [6]. Moreover, in February 2019 the French National Reference Center for Arbovirus diagnosed a pregnant woman who returned from Thailand infected with Zika virus (unpublished data, positive Zika RT-PCR and positive serum IgM antibodies to Zika virus using methods described in the appendix of [7]).

In conclusion, RDTs for dengue require standardised evaluation and must be validated in the epidemiological context of their use, especially in tropical and subtropical regions where various vector-borne pathogens may circulate.
